# Free light chains: potential biomarker and predictor of mortality in alpha-1-antitrypsin deficiency and usual COPD

**DOI:** 10.1186/s12931-016-0348-1

**Published:** 2016-03-31

**Authors:** Judith A. Hampson, Robert A. Stockley, Alice M. Turner

**Affiliations:** Centre for Translational Inflammation Research, University of Birmingham, Birmingham, B15 2WB UK; ADAPT Project, University Hospital Birmingham, Birmingham, B15 2WB UK; Heart of England NHS Foundation Trust, Birmingham, B9 5SS UK

**Keywords:** Chronic obstructive pulmonary disease, Emphysema, Alpha 1 antitrypsin deficiency, Mortality, Exacerbations, Immunoglobulin light chains

## Abstract

**Background:**

Circulating free light chains (FLCs) can alter neutrophil migration, apoptosis and activation and may be a biomarker of autoimmune disease and adaptive immune system activation. These pathogenic roles could be relevant to lung disease in alpha 1 antitrypsin deficiency (A1ATD) and chronic obstructive pulmonary disease (COPD).

**Methods:**

Total combined (c)FLCs were measured using the FreeLite® assay in 547 patients with A1ATD and 327 patients with usual COPD in the stable state, and assessed for association with clinical phenotype, disease severity, airway bacterial colonisation and mortality. Univariate and multivariate analyses were undertaken.

**Results:**

Circulating cFLCs were static in the stable state when measured on 4 occasions in A1ATD and twice in usual COPD. Levels were inversely related to renal function (A1ATD and COPD *p* = <0.01), and higher in patients with chronic bronchitis (*p* = 0.019) and airway bacterial colonisation (*p* = 0.008). After adjusting for renal function and age the relationship between cFLCs and lung function was weak. Kaplan Meier curves showed that cFLC > normal (43.3 mg/L) significantly associated with mortality in both cohorts (A1ATD *p* = 0.001, COPD *p* = 0.013).

**Conclusions:**

cFLCs may be a promising biomarker for risk stratification in A1ATD and COPD.

**Electronic supplementary material:**

The online version of this article (doi:10.1186/s12931-016-0348-1) contains supplementary material, which is available to authorized users.

## At a glance commentary

### Scientific knowledge on the subject

Circulating free light chains are a potential biomarker of adaptive immune activation, which influence neutrophil function and are elevated in an animal model of emphysema. The ADAPT and WMCC cohorts received ethical approval from the South Birmingham National Research Ethics Service (NRES) committee (ADAPT ethics approval number 3359a, WMCC REC ref no. 07/H1207/231). The IRF Usual COPD cohort received ethical approval from the East Midlands NRES committee (REC ref no.12/EM/0090).

### What this study adds to the field

Circulating free light chains relate to clinical phenotype and prognosis in both A1ATD and usual COPD, and are elevated in the context of a possible stimulus to the adaptive immune system, namely chronic bacterial colonization of the airway.

## Background

Chronic obstructive pulmonary disease (COPD) is an inflammatory lung disease characterised by persistent airflow obstruction, which usually progresses [1]. Immune activation may be one of the factors perpetuating inflammation in COPD [2]. The immune response seen in COPD incorporates cells from innate and adaptive immune systems [3]; an essential component of adaptive immunity is the production of antibodies by mature B lymphocytes. Antibodies are immunoglobulins which are composed of two polypeptide heavy chains and two light chains. During antibody production there is an excess of free light chains (FLCs) produced which are secreted into the circulation before undergoing renal clearance [4]. There are two free light chain isotypes: kappa (κ) and lambda (λ), which can be measured by a highly sensitive assay [5]. High FLC levels occur in several autoimmune and inflammatory conditions, thus suggesting they may be a biomarker of adaptive immune activation [4]. Raised polyclonal FLCs have also been reported in a number of respiratory conditions where adaptive immunity may be important, including asthma [6] and COPD [7].

In addition to being a marker of immune activation, FLCs could have a direct pathogenic role in COPD. They inhibit neutrophil apoptosis [8], inhibit neutrophil migration in vitro [9], and are elevated in both murine models of emphysema and serum from patients with COPD [7]. In addition FLCs bind to human neutrophils, activating them to produce IL8 in vitro; specific FLC antagonism inhibited this binding capability and reduced pulmonary neutrophilia in smoke exposed mice [7].

The primary aim of this study was to investigate the clinical utility of FLCs as a biomarker in patients with alpha-1-antitrypsin deficiency (A1ATD). We hypothesised that FLCs would be static in stable disease, relate to disease severity, distinguish clinically relevant subgroups, relate to factors which could stimulate the adaptive immune system and associate with longitudinal outcomes, such as mortality. We then sought to replicate these associations in “usual” (non A1ATD) COPD. Furthermore we hypothesised that levels would be similar in usual COPD to A1ATD, since pulmonary immune activation has recently been shown to be similar between these groups [10].

## Methods

### Study design and population

Patients with severe A1ATD (defined as a level of <11 μM and phenotype PiZZ, PiZnull or other dysfunctional variants) were selected from the UK A1ATD registry (*n* = 547). Recruitment and follow up procedures are described in detail elsewhere [11]; briefly patients attend annually for review, and are followed up until death. Lung function has been calculated on all patients with at least 3 years of serial lung function [12]. The usual COPD cohort (*n* = 327) comprised all patients in the West Midlands COPD Collection (WMCC), recruitment procedures for which are described elsewhere [13], and a second cohort recruited through the Inflammation Research Facility (IRF) at Queen Elizabeth Hospital. The IRF cohort has superceded the WMCC and largely replicates assessment procedures of the UK A1ATD registry, again following up annually until death. Both cohorts were reviewed in the stable state only, thus were only suitable for repeated measurement of cFLC levels in stable disease, cross-sectional analyses of clinical phenotype (including relevant co-morbidities) and survival analysis. All studies were approved by the local ethics committee and patients gave informed consent.

Baseline demographic data including age, sex and pack years smoked was collated for all cohorts. CT scans were examined for evidence of emphysema and bronchiectasis and post-bronchodilator lung function recorded. Symptom history (e.g., presence of chronic bronchitis), annual exacerbation frequency and mortality were noted. The exacerbation history was assessed using the criteria suggested by Anthonisen et al. of increased breathlessness, sputum volume and sputum purulence [14]. In addition, we recorded patients’ renal function as measured by their creatinine and urea, and calculated the estimated glomerular filtration rate (eGFR) [15]. In the A1ATD cohort, stable state quantitative sputum culture results were examined to establish if patients were chronically colonised with pathogens (≥1x10^5^ cfu/ml in ≥3 samples over ≥3 months [16]). Although patients with stable COPD were asked to provide sputum samples, identical to the A1ATD group, there were too few for meaningful analysis.

### FLC analysis

The FLC analysis was performed on serum or plasma in all patients using the Freelite® immunoassay (Binding site Group Ltd, Birmingham, UK), summating κ and λ values to give a combined (c)FLC result, as it is the combined level which has been of relevance in past studies of other diseases and in the general population. Reference ranges were combined (κ&λ) FLC (cFLC) 9.3–43.3 mg/L [17] and κ/λ ratio 0.26–1.65 [18]. Patients with an abnormal κ/λ ratio (suggestive of possible underlying monoclonal gammopathy) were excluded from analysis. We conducted experiments using matched serum and plasma samples demonstrating equivalence of measures in the two sample types (Additional file 1). In 19 A1ATD patients and 59 usual COPD patients with stable disease (according to both history and serial lung function) we performed FLC analysis on ≥2 samples collected over a period ranging from 1 to 4 years to assess whether levels were static in stable patients.

### Statistical analysis

All analyses were performed using IBM SPSS statistics version 20. Data distribution was assessed and univariate analyses performed using Mann Whitney U tests or Spearmen’s Rho (r_s_) correlations; results are presented as median (IQR). Relationships between cFLC and all variables shown in Table 1 were examined in A1ATD and usual COPD, as were current smoke exposure and airway bacterial colonisation (potential immune activation triggers). Multivariate analytic techniques were then used to adjust for covariates where needed; renal function (eGFR) was included in all regression analyses. A1ATD and COPD were subsequently compared using logistic regression, including as covariates eGFR and those factors relating to cFLC levels that differed between the 2 cohorts. The Friedman test was used to establish whether cFLC measures were static in stable patients. For the mortality analyses survival time was defined as time from date of sample collection to analysis date; univariate and multivariate Cox regression analyses were performed, selecting regression covariates if *p* < 0.1 in the univariate analysis, and no significant collinearity with other variables. Cox regression was conducted using cFLC as a continuous variable and also using 2 important threshold levels – the upper limit of normal (43.3 mg/L) and the threshold associated with death within 100 days (65 mg/L [19]). Kaplan – Meier curves were drawn to compare these thresholds.

**Table 1:** Characteristics of the A1ATD and usual COPD cohorts

Variable		A1ATD cohort (*n* = 540)	Usual COPD cohort (*n* = 316)	*p*
Age		53.9 (45.0–60.9)	68.8 (61.5–75.1)	**<0.001**
Sex		Male *n* = 311 (57.6 %)	Male *n* = 182 (57.6 %)	0.999
		Female *n* = 229 (42.4 %)	Female *n* = 134 (42.4 %)	
Pack years		11.6 (0.0–24.0)	44.1 (29.5–62.2)	**<0.001**
FEV1 (% predicted)		50.7 (35.1–85.1)	46.4 (35.0–61.0)	**<0.001**
KCO (% predicted)		62.3 (49.3–77.0)	59 (47.5–77.0)	0.533
Chronic bronchitis		185 (34.3 %)	198 (62.7 %)	**<0.001**
Emphysema		358 (66.3 %)	257 (81.3 %)	**0.002**
Bronchiectasis		142 (26.3 %)	96 (30.3 %)	0.678
Frequent exacerbator		129 (40.8 %)	193 (61.1 %)	**<0.001**
eGFR		81.3 (70.6–93.9)	85.8 (69.7–101.1)	0.063
CKD stage				**0.002**
1	eGFR ≥ 90	156 (28.9 %)	119 (37.6 %)	
2	eGFR 60–89	279 (51.7 %)	130 (41.1 %)	
3	eGFR 30–59	41 (7.6 %)	35 (11.1 %)	
4	eGFR 15–29	2 (0.4 %)	4 (1.3 %)	
5	eGFR < 15	1 (0.2 %)	0 (0 %)	
Unknown (no eGFR)		61 (11.3 %)	28 (8.9 %)	
cFLC (mg/L)		25.7 (21.1–31.7)	31.9 (24.0–43.3)	**<0.001**
κ/λ		0.86 (0.71–1.08)	0.86 (0.72–1.06)	0.967

Number of patients with contemporaneous renal function = 479 A1ATD, 288 usual COPD. Continuous variables expressed as median (IQR); sex, chronic bronchitis, emphysema, bronchiectasis, exacerbators and CKD stage expressed as number in each group (%). A frequent exacerbator was defined as having 2 or more exacerbations per year. *BMI* body mass index, *FEV1* forced expiratory volume in 1 s, *KCO* corrected gas transfer, *eGFR* estimated glomerular filtration rate, *cFLC* combined (κ + λ) free light chain level (mg/L)

*P* values highlighted in bold indicate significant differences between the A1ATD and Usual COPD cohorts (*p* ≤ 0.05)

## Results

### Stable A1ATD

Median follow up time was 5.7 (3.9–7.7) years. The demographics of the cohort are outlined in Table 1. Eighty four percent of patients had post-bronchodilator airflow obstruction (defined as an FEV1/FVC ratio <0.7); 8 of those without obstruction had emphysema on CT scan. Seven patients were excluded due to an abnormal κ/λ ratio. At least one autoimmune disease was present in 15.6 % of patients (*n* = 84), the most common being thyroid disease (4.1 %, *n* = 22), diabetes (3.1 %, *n* = 17), ulcerative colitis (2.1 %, *n* = 11), psoriasis (1.3 %, *n* = 7) and vasculitis (0.9 %, *n* = 5). cFLC levels did not differ between patients with and without autoimmune disease (*p* = 0.125) and there was no difference in the number of autoimmune conditions exhibited by patients with cFLC levels outside the normal range (>43.3 mg/L; *p* = 0.320) compared to those with normal levels. There was no significant difference in cFLCs taken at 4 time points in stable disease (*p* = 0.116).

#### Cross sectional univariate analyses of COPD phenotype and clinical features against cFLC

**Fig. 1:**
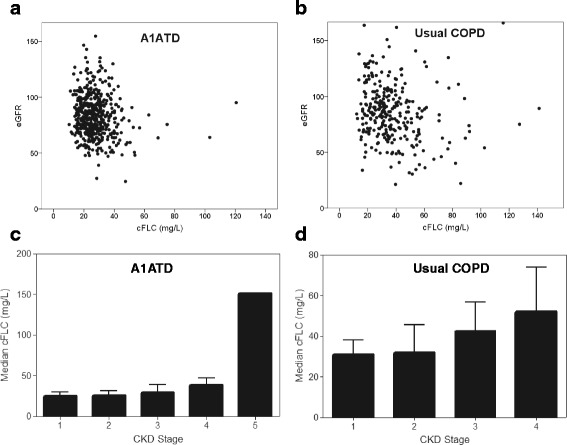
Stable state cFLC levels relate to renal function. Scatter plots (**a**, **b**) and bar charts (**c**, **d**) showing the relationship between cFLC (mg/L), eGFR (ml/min/1.73 m^2^) and chronic kidney disease (CKD) stage in the stable state in A1ATD and usual COPD patients respectively. *P* values are for 2 tailed univariate statistics. A1ATD (a) r_s_ = −0.14, *p* = 0.003 (c) *p* = 0.006. Usual COPD (b) r_s_ = −0.24, *p* = <0.001 (d) *p* = <0.001. A1ATD CKD stage 1 (*n* = 156), 2 (*n* = 279), 3 (*n* = 41), 4 (*n* = 2), 5 (*n* = 1). Usual COPD CKD stage 1 (*n* = 119), 2 (*n* = 130), 3 (*n* = 35), 4 (*n* = 4), 5 (*n* = 0)

There was a weak negative correlation between cFLC level and eGFR (r_s_ = −0.14, *p* = 0.003), consistent with the difference seen between CKD stage groups (*p* = 0.006; Fig. 1). Age also correlated with cFLCs (r_s_ = 0.15, *p* = 0.001), but this relationship disappeared after adjustment for eGFR r_s_ = 0.08, *p* = 0.101), suggesting this was primarily due to worsening renal function with age. Given the renal clearance of FLCs the relationship to eGFR was expected but important to confirm.

Analyses controlling for eGFR and age demonstrated weak correlations between cFLC and lung function (FEV1 *r* = 0.13, *p* = 0.012, KCO *r* = 0.10, *p* = 0.046). No significant differences were seen with respect to presence of bronchiectasis or emphysema, or frequent exacerbations (defined as ≥2 per year). Patients with chronic bronchitis had significantly higher cFLCs compared to those without (27.0 (21.1–33.6) v 25.0 (20.8–30.8); *p* = 0.019).

**Fig. 2:**
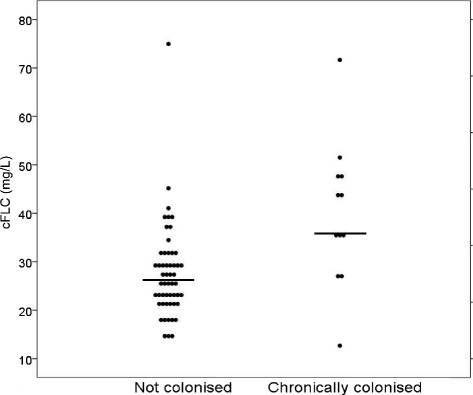
Relationship of cFLC levels to airway bacterial colonisation. The graph shows a univariate comparison between colonised and non-colonised individuals with A1ATD, each dot represents a patient, and the horizontal line is the median per group, *p* = 0.008. (cFLC = combined κ & λ mg/L)

No significant relationships were seen between cFLC and smoke exposure (either current smoking status or pack years). Sputum specimens during stable disease were available in 152 patients. Of these, 53 had no positive sputum cultures and 12 were chronically colonised with ≥1 potentially pathogenic organism. Patients who were chronically colonised had significantly higher cFLCs compared to patients with no positive cultures (Fig. 2; 35.7 (26.4–42.4) v 26.3 (22.0–31.2) *p* = 0.008).

#### Longitudinal outcomes and cFLC levels

**Fig. 3:**
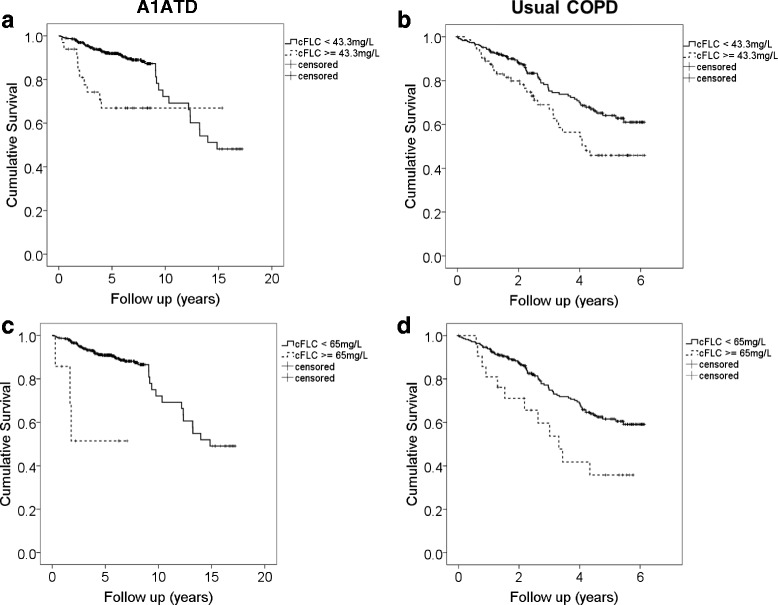
Kaplan-Meier curves exploring the cut-off level of cFLC which relates to death. Kaplan-Meier survival curves according to cFLC levels in the A1ATD and usual COPD cohorts, solid lines representing those with levels below the specified threshold and dotted lines those with a level above it. A1ATD cohort ≥43.3 mg/L (*n* = 33), <43.3 mg/L (*n* = 507), ≥65 mg/L (*n* = 7), <65 mg/L (*n* = 533). Usual COPD cohort ≥43.3 mg/L (*n* = 79), <43.3 mg/L (*n* = 237), ≥65 mg/L (*n* = 24), <65 mg/L (*n* = 292). Please note the differing time scales, due to longer duration of follow up in A1ATD. All statistics are by log-rank test, and plots are unadjusted for covariates. A1ATD **a** Threshold of normal range (43.3 mg/L) *p* = 0.001 **c** Threshold ≥65 mg/L *p* <0.001. Usual COPD **b** Threshold of normal range *p* = 0.013 **d** Threshold ≥65 mg/L *p* = 0.012

Sixty nine (12.8 %) patients died. Patients who died had significantly higher baseline cFLCs compared to those who remained alive (29.2 (22.7–39.9) v 25.2 (21.0–31.0), *p* = 0.001). Multivariate analysis by Cox regression, to assess whether cFLC associated with mortality, showed that cFLC, increasing age and lower FEV1 (all *p* < 0.001) significantly predicted death, with a cFLC level above the normal range conferring an odds ratio for death of 2.89 (1.47–5.70) *p* = 0.002. The Kaplan-Meier plots showed significant differences in the survival curves according to both this level and the higher figure of 65 mg/L (Fig. 3). However cFLCs were not a sufficiently sensitive or specific test to perform well in ROC curves plotted against mortality (AUC for either threshold <0.62).

### Stable usual COPD

The median follow up time was 2.5 (1.5–4.7) years. Clinical features and demographics are shown in Table 1. Eleven patients were excluded due to an abnormal κ/λ ratio.

As in A1ATD levels remained static in stable disease (*p* = 0.153). There was a similar relationship between cFLC level, eGFR and CKD stages to that observed in A1ATD (Fig. 1), although the degree of variability in usual COPD was greater than A1ATD, perhaps due to higher clinical heterogeneity of usual COPD compared to A1ATD. The correlation between cFLC and eGFR was also reduced by adjustment for age, but did not disappear. Unlike A1ATD, men had significantly higher cFLC levels than women (35.4 (27.4–47.2) v 29.51 (21.6–38.8), *p* < 0.001). Similar regression analyses to A1ATD were performed, with the addition of sex as a covariate. Higher cFLC levels also tended to occur with chronic bronchitis (34.1 v 32.3; *p* = 0.087) and the lack of relationship to bronchiectasis and emphysema persisted.

#### Longitudinal outcomes and cFLC levels

Ninety one (28.8 %) patients died. The relationship between higher cFLCs and subsequent death was replicated in usual COPD (36.4 (26.0–51.5) v31.5 (23.3–41.3), *p* = 0.014), and the multivariate model confirmed that cFLC remained independently associated with mortality, with a level above the normal range conferring an odds ratio of 1.80 (1.16–2.80), *p* = 0.009. Similar patterns were seen in the Kaplan-Meier curves (Fig. 3).

### Comparison of A1ATD and usual COPD

The primary purpose of this analysis was to compare the cFLC levels between the 2 groups, rather than their clinical features, as cohorts were recruited in different settings, and differences between A1ATD and usual COPD are well known. Since cFLC related to several clinical features in both A1ATD and usual COPD it was necessary to adjust by regression analysis for these features prior to comparing cFLC levels; this was particularly important because most of these also differed between A1ATD and our usual COPD cohort (Table 1). This was achieved by forced entry of eGFR, FEV1 % predicted, presence of chronic bronchitis and age as covariates in a linear regression seeking associations of cFLC levels, to which the presence of A1ATD was then added as a final variable. Since cFLC were non-normally distributed the values were logged prior to regression analysis, which then resulted in normal distribution of the subsequent standard residuals.

Combined free light chain levels appeared higher in usual COPD than A1ATD (Table 1). A1ATD was a significant predictor of lower cFLCs independent of eGFR, FEV1, chronic bronchitis and age (*p* < 0.001; Table 2).

**Table 2:** Linear regression analysing log cFLC in all stable patients

Variable	B (95 % CI)	R^2^ change	F change	*P* (F change)
eGFR	−0.001 (−0.002–−0.001)	0.04	19.94	<0.001
Age	0.001 (0–0.003)	0.03	16.40	<0.001
Chronic bronchitis	0.033 (0.007–0.06)	0.02	9.72	0.002
FEV1% predicted	4.8 × 10^−4^ (0–0.001)	0.01	3.00	0.084
A1ATD	−0.09 (−0.133–−0.053)	0.03	21.10	<0.001

The table shows the regression coefficients (B) and significance of variables. The two most important variables in the model were eGFR and A1ATD

## Discussion

Our main objective was to investigate the utility of measuring polyclonal FLCs as a clinical biomarker in severe A1ATD and usual COPD. Key properties of a clinically useful biomarker are that it is reproducible in stable disease, relates to disease severity and relates to outcome. Our results demonstrate that cFLCs meet many of these criteria, notably being associated with subsequent mortality in both our cohorts. No significant difference was seen in cFLCs taken from patients with stable disease at different time points, suggesting that cFLCs are reproducible in stable disease. We did not see a strong relationship between cFLC levels and disease severity, although there was a difference observed between patients with and without chronic bronchitis, which is recognised to be a clinically relevant subgroup within airways disease [20].

A role for the adaptive immune system in perpetuation of inflammation in COPD has been proposed, since accumulation of B cells in large and small airways associates with worsening disease severity [21]. FLCs, produced as a by-product of immunoglobulin synthesis by mature B cells, could be a useful marker of adaptive immune system activity [4]. The prevalence of other autoimmune diseases was low in our A1ATD cohort, and no relationships were seen between cFLC levels and autoimmune disease burden. However, prior studies suggest that cFLCs change during periods of disease ‘activity’ (e.g. in rheumatoid arthritis [22] and systemic lupus erythmatosus [23]) such that presence of well controlled (inactive) autoimmune conditions might explain the lack of association observed. Furthermore, many important questions regarding the role B cells play in the development of COPD remain unanswered. For example, which antigens drive the B cell response? Is the response specific to the lung or not? If it were lung specific, then this might account for the lack of relationship to co-morbid systemic diseases linked to immune activation. Commonly hypothesised antigen sources are microbes colonising the airways, smoke constituents and breakdown products of the extracellular matrix [24]. In the A1ATD cohort we found that chronically colonised patients had significantly higher cFLC levels, supporting the hypothesis that colonisation may be an important driving force behind adaptive immune activation.

Another theory is that infection or colonisation with bacteria leads to a breakdown in self – tolerance promoting an immune reacton to self-antigens. This concept is well established in a number of autoimmune diseases [25] and there is some evidence supporting an autoimmune element to COPD [2]. The difference in cFLC observed between usual COPD and A1ATD imply that this is a more important pathogenic theme in usual COPD, although this does not exclude immune activation contributing to the disease process in A1ATD. This result is contrary to the recent report of equivalent levels of lymphoid follicles in lung tissue from a small cohort of A1ATD patients with very severe lung disease, compared to usual COPD [10]. It is possible that immune activation represents a feature of advanced disease in both conditions, as most of our patients had severe disease, thus further studies are indicated.

Mechanistically cFLCs have biological properties that could potentially damage lung tissue through interaction with neutrophils [7–9]. We have shown previously that migratory accuracy of neutrophils is lower in COPD than A1ATD [26]; it is possible that cFLCs might be partly responsible since cFLCs were significantly higher in COPD in our study. Furthermore several case reports detail nodular and cystic lung disease associated with cFLC overproduction in light chain deposition disease (LCDD) [27–30], which is characterised by the deposition of non-amyloid κ or λ light chains, and presents with progressive cystic lung disease ultimately leading to respiratory failure necessitating lung transplantation [29]. It is possible to therapeutically antagonise cFLCs using the compound F991 in animals [6, 7, 31]. Thus it remains important to clarify whether the pro-inflammatory effects of cFLCs play a role in COPD, and thus represent a suitable drug target.

Our results also demonstrated that patients with chronic bronchitis had significantly higher cFLCs, suggestive of a greater adaptive immune response in these individuals. Chronic bronchitis is associated with more rapid FEV_1_ decline [32], increased exacerbation frequency [33] and a greater risk of mortality [34]. However, the difference between cFLCs in patients with chronic bronchitis compared to those without was relatively small, thus the result must be interpreted with caution in terms of clinical significance. The sex difference in cFLCs in usual COPD was unexpected. There is known to be a male predominance in haematological conditions associated with monoclonal overproduction of FLCs such as monoclonal gammopathy of unknown significance (MGUS) and multiple myeloma [35] however in our study, any patients with an abnormal κ/λ were exluded from the analysis, hence undetected gammopathies would not have influenced our results.

Finally, we have shown that raised circulating cFLC levels are a predictor of mortality, independent of age and severity of renal impairment. Several studies have shown a link between immune system activity, inflammation and risk of death: an increase in polyclonal cFLCs predicted mortality in the general population [17] and cFLC >65 mg/L were a risk factor for death within 100 days [19]. The association between inflammation and cardiovascular death is well reported [36], and 41 % of the deaths in those with cFLC > 65 mg/L were from cardiovascular disease [17]. A recent systematic review supported the concept that the relationship between cardiovascular disease and COPD goes beyond common aetiological factors such as smoking [37]; cFLCs could partly explain this.

Our study is limited by the relative lack of colonisation data in A1ATD and absence of this information in usual COPD. Our A1ATD cohort is similar in disease severity to the American A1ATD registry [38], thus results are likely to be generalisable to other A1ATD populations. However the usual COPD group generally had severe COPD, and exhibited high prevalence of emphysema, thus our results may be less generalisable to milder usual COPD cohorts. The severity of their disease is highlighted by the significant differences relative to A1ATD, who in many cases appear less unwell. This is in part due to the inclusion of family screened, non-index cases in A1ATD, but not usual COPD. It would also be of interest to measure cFLCs in an adequate number of exacerbations of COPD, to see if flare ups of disease relate to cFLC levels, as they do in some autoimmune diseases. Sample collection for this is now ongoing.

## Conclusions

To our knowledge this is the first study to evaluate the utility of cFLCs as a clinical biomarker in A1ATD and usual COPD. Elevated FLCs independently predict mortality in patients with severe A1ATD and usual COPD and could play a role in risk-stratification of patients requiring more intensive monitoring and management.

## Additional file

Additional file 1: Plasma and serum FLC matched samples analysis: methods and results. (DOCX 30 kb)
