# Supplementary material for: Free light chains: potential biomarker and predictor of mortality in alpha-1-antitrypsin deficiency and usual COPD
Source: Respir Res. 2016 Mar 31;17:34. doi: 10.1186/s12931-016-0348-1 (PMC4815123; doi:10.1186/s12931-016-0348-1)
Supplement: Additional file 1: — Plasma and serum FLC matched samples analysis: methods and results. (DOCX 30 kb) [file 12931_2016_348_MOESM1_ESM.docx]

**Online supplement**

**Plasma and serum FLC matched samples analysis: methods and results**

In a subgroup of 82 patients from the usual COPD cohort paired serum and plasma samples were analysed. Samples were collected from patients and assayed using Freelite® (The Binding Site Group Ltd (TBS), Birmingham, UK) on the SPAPLUS® turbidimeter (TBS). 3 patients were excluded from the analysis due to an abnormal κ/λ ratio. Statistical analysis was performed using Analyse-It® and Prism v5®. There was a very high correlation between serum and plasma κ and λ values (Spearman’s Rho: 0.96 and 0.98 respectively, p <0.001), and consequently also between cFLC levels (e-figure 1, below). The results from both matrices were also equivalent using Passing-Bablok analysis: κ: y=0.94x -0.32 and linear regression analysis: y=0.90x - 0.5 and λ: y=0.92x - 0.11; y=0.92x - 0.05. The assay also showed good agreement whether plasma or serum samples were tested: κ: PPV: 89%, NPV: 93% and λ: PPV: 100%, NPV: 93%. The results therefore indicate good analytical agreement between FLC values determined using the Freelite assay in both plasma and serum.


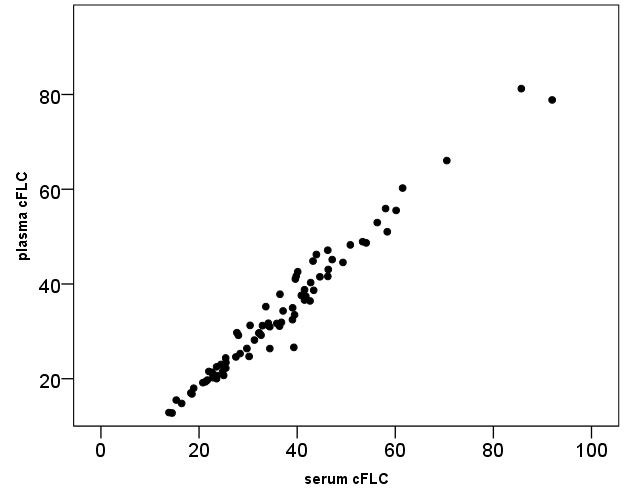


E-Figure 1: Correlation between serum and plasma cFLC levels

N=79, σ=0.996

Effect of inclusion of plasma samples on comparison A1ATD and usual COPD

All A1ATD cFLC measurements were made on serum. 82 of the usual COPD cohort had both serum and plasma available, and the remainder had either serum or plasma. Whilst values for serum and plasma were equivalent on the assay we wished to exclude the possibility that small sample differences might have affected the comparison between groups, as the COPD group contained some measurements made solely on plasma. We therefore excluded the patients whose measurements were only made on plasma and repeated our analyses. Patients with A1ATD had lower levels of serum cFLC, as in the analysis using either plasma or serum (median 25.69 v 34.42; p<0.0001), a result which was maintained in the multiple linear regression, containing eGFR, Age, FEV1 % predicted and chronic bronchitis as covariates, and analysing log cFLC as before. A1ATD remained associated with lower cFLC (p<0.0001).
